# Full-Length ASFV B646L Gene Sequencing by Nanopore Offers a Simple and Rapid Approach for Identifying ASFV Genotypes

**DOI:** 10.3390/v16101522

**Published:** 2024-09-26

**Authors:** Vivian O’Donnell, Edward Spinard, Lizhe Xu, Amy Berninger, Roger W. Barrette, Douglas P. Gladue, Bonto Faburay

**Affiliations:** 1 Foreign Animal Disease Diagnostic Laboratory, National Veterinary Services Laboratories, Animal and Plant Health Inspection Service, U.S. Department of Agriculture, Plum Island Animal Disease Center, Orient, NY 11944, USA; vivian.odonnell@usda.gov (V.O.); lizhe.xu@usda.gov (L.X.); roger.w.barrette@usda.gov (R.W.B.); 2 Agricultural Research Service, U.S. Department of Agriculture, Plum Island Animal Disease Center, Orient, NY 11944, USA; edward.spinard@usda.gov (E.S.); doug@seeklabs.com (D.P.G.); 3 National Bio- and Agro-Defense Facility (NBAF), Agricultural Research Service, U.S. Department of Agriculture, Manhattan, KS 66502, USA; 4 Oak Ridge Institute for Science and Education (ORISE), Oak Ridge, TN 37831, USA; amy.berninger@usda.gov

**Keywords:** African swine fever, p72, Nanopore, next-generation sequencing, genotyping

## Abstract

African swine fever (ASF) is an acute, highly hemorrhagic viral disease in domestic pigs and wild boars. The disease is caused by African swine fever virus, a double stranded DNA virus of the *Asfarviridae* family. ASF can be classified into 25 different genotypes, based on a 478 bp fragment corresponding to the C-terminal sequence of the B646L gene, which is highly conserved among strains and encodes the major capsid protein p72. The C-terminal end of p72 has been used as a PCR target for quick diagnosis of ASF, and its characterization remains the first approach for epidemiological tracking and identification of the origin of ASF in outbreak investigations. Recently, a new classification of ASF, based on the complete sequence of p72, reduced the 25 genotypes into only six genotypes; therefore, it is necessary to have the capability to sequence the full-length B646L gene (p72) in a rapid manner for quick genotype characterization. Here, we evaluate the use of an amplicon approach targeting the whole B646L gene, coupled with nanopore sequencing in a multiplex format using Flongle flow cells, as an easy, low cost, and rapid method for the characterization and genotyping of ASF in real-time.

## 1. Introduction

African swine fever (ASF) is a highly contagious and often lethal hemorrhagic viral disease of domestic pigs and wild boars. Outbreaks of the disease can have significant economic consequences for the swine industry, including loss of animals and restrictions of export markets to countries who are free of the disease. The causative agent, ASF virus (ASFV), is currently the only member of the *Asfarviridae* family. ASFV is an enveloped virus, with a double-stranded DNA genome of approximately 190 kbp encoding 150–200 genes [1]. First described in Kenya in 1921, ASF outbreaks have been occurring in sub-Saharan Africa ever since, with occasional outbreaks outside of Africa, that were resolved; however, in 2007 a single outbreak of a genotype II strain occurred in the Republic of Georgia, resulting in a pandemic with continued outbreaks in Europe, Asia, and most recently on the island of Hispaniola [2,3,4,5,6].

Due to the high conservation of the p72 coding region, Bastos et al., demonstrated the usefulness of genotyping based on the partial B646L nucleotide sequence of the p72 C-terminal region and its suitability for determining the viral relationships of field strains [7]. However, recent efforts have determined that a full-length B646L sequence would be a better source for genotyping ASFV by p72 [8], reducing the 25 genotypes into only six genotypes. Furthermore, the authors demonstrated that partial sequencing of p72 may fail to identify the correct genotype [8]. As p72 has been historically used for epidemiological tracking of different field isolates, as well as genotype assignment through phylogenetic trees, then, it is of interest to have the capability to sequence the full-length B646L gene (p72) for a quick and accurate genotype characterization. Nanopore sequencing has been previously described for identification of ASFV and other infectious diseases using an amplicon approach, targeting a small region of the B646L gene [9,10]. However, the current primers for genotyping by p72 are not suitable for full-length B646L sequencing because they only cover the C-terminal region of this gene, so new primers are needed. Here we describe a set of primers that can be used for full-length B646L sequencing.

Understanding the circulating strains of ASFV is of great importance for characterizing disease outbreaks and implementing appropriate control measures. As Vietnam is the only country to approve vaccination, control of the disease in outbreak situations and in endemic areas is highly dependent on rapid pathogen detection and characterization. The recent reports of genotype I and recombinant genotype I/II strains circulating in Asia [11,12] have emphasized the need to characterize ASFV by whole genome sequencing and biotyping rather than classification by a single gene [13]. Still, whole genome sequencing is not feasible for routine application in most laboratories in endemic and outbreak areas or for application in large scale surveillance of ASFV. Furthermore, although whole genome sequencing may provide detailed information, it is technically demanding and time-consuming, requiring implementation of lengthy protocols, and requires higher computational power to resolve the sequences. Consequently, a simple and rapid genotyping method for ASFV is urgently needed.

In this study, we propose the use of a pool of primers for the whole B646L gene as an easy and rapid method for the characterization of ASFV. The approach will not require significant methodological changes or extra effort for laboratories that are already implementing ASFV genotyping using partial B646L sequences, which often can be sequenced and characterized by traditional Sanger methods or by next generation sequencing (NGS) technology. In this work, we evaluated the use of a set of primers homologous to a region outside B646L (p72) that were designed to synthesize a PCR product containing the entire length of B646L (p72), which can then be used for targeted amplicon sequencing. These primers were designed from the conserved regions of ASFV sequences currently available in GenBank. Here, we applied a PCR method to generate a full-length B646L (p72) target amplicon in less than 2 h, coupled with Nanopore sequencing, to sequence in real time the whole B646L gene. We evaluated both fresh and frozen samples, which are typical sample types submitted to a diagnostic laboratory, for determining if suspect samples were positive for ASF in an outbreak situation.

## 2. Materials and Methods

### 2.1. Primer Design

Two forward primers located at positions 139 and 150 upstream (corresponding to nucleotide positions 104,398 and 104,441 on the genome of Georgia/2007/1, accession FR682468.2) of B646L (p72) and two reverse primers located at positions 113 and 150 downstream (corresponding to nucleotide positions 106,650 and 106,668 on Georgia/2007/1’s genome) of the B646L gene (p72) were designed to focus on conserved areas upstream or downstream of B646L gene. All primer sequences were compared against 260 full-length ASFV genome sequences using BLASTN with the following parameters: task = blastn-short, max_hsps = 1, and max_target_seqs = 4. Exact matches and the location of SNPs were then identified and quantified (Primer alignment on ASF genome flanking p72 and Supplementary Table S1—Excel File). All primers were synthesized (Integrated DNA Technologies, Coralville, IA, USA), reconstituted to 100 µM, and stored at −20 °C for further evaluation (Table 1).

### 2.2. ASF Strains Used to Evaluate the Set of Primers for B646L (p72)

Initial analysis of the primer pairs was done with six ASFV strains: Georgia 2007/1, Lisbon/60, Killean III, Kimakia-64, Malawi Lil-20/1, and Pretoria-4/1, provided by the U.S. Department of Agriculture, National Veterinary Services Laboratories’ Foreign Animal Disease Diagnostic Laboratory (USDA-NVSL-FADDL), Plum Island, NY, USA. For this experiment, primer pairs were evaluated in all four possible combinations and as a pool of all four primers. Furthermore, DNA extracted from a panel of 19 ASFV strains corresponding to different genotypes, provided by USDA-NVSL-FADDL, Plum Island, NY, USA, was used for evaluation of the four pooled primers (Table 2).

### 2.3. Porcine Whole-Blood Samples for the Detection of the ASFV B646L Gene (p72)

Blood samples (EDTA stabilized) were collected from five pigs experimentally infected by intramuscular administration with 10^5^ TCID_50_/mL of a genotype II representative strain, ASFV Georgia 2007/1, or 10^5.4^ TCID_50_/mL of a genotype I representative strain, ASFV Lisbon/60. Blood samples were collected from three ASFV Georgia 2007/1-infected pigs 6 days post inoculation (d.p.i.), and from two ASFV Lisbon/60-infected pigs at 7 d.p.i. and at 9 d.p.i. All samples were taken when clinical signs of disease were observed and processed immediately following collection (fresh samples). Further, an aliquot of each sample was stored at −70 °C (frozen samples) for subsequent analysis. All samples were provided by the Foreign Animal Disease Diagnostician (FADD) Training Course, USDA-NVSL-FADDL, Plum Island, NY, USA (Table 3).

### 2.4. ASFV Nucleic Acid Extraction

Total nucleic acid was extracted from all samples using the MagMax Core kit (Thermo Fisher Scientific, Waltham, MA, USA) on a 96-well magnetic bead robotic platform (Applied Biosystems, Woburn, MA, USA), following the manufacturer’s protocol. Extracted nucleic acids were transferred to LoBind tubes, quantified using Qubit 1X dsDNA high sensitivity kit (Thermo Fisher Scientific, Waltham, MA, USA), and used for PCR, to generate B646L (p72) amplicons, and to prepare the subsequent library.

### 2.5. Detection of ASFV by Real-Time PCR

Detection of ASFV in all samples was performed using specific primers and a probe to amplify the C-terminal end of the ASFV p72 major capsid protein, as a modification of Zsak et al. [14]. Each 25 µL reaction mixture contained 2.5 µL of nucleic acid template, 1.25 µL of primer-probe mix (forward: CTT Cgg CgA gCg CTT TAT CAC, reverse: ggA AAT TCA TTC ACC AAA TCC TT, probe: FAM–CgA TgC AAg CTT TAT–MGB/NFQ), 6.25 µL of enzyme mix (TaqMan^®^ Fast Virus 1-Step Master Mix, Thermo Fisher), and 15 µL of nuclease-free water. Real-time PCR (qPCR) was performed using a real-time PCR system (ABI7500, Thermo Fisher). Cycling conditions consisted of denaturation at 95 °C for 20 s for 1 cycle, 45 cycles of amplification at 95 °C for 10 s, and 60 °C for 30 s in standard run mode. Samples with a threshold cycle (CT) value equal to or less than 40 were considered positive, which is consistent with the NVSL FADDL testing algorithm for ASFV [15]

### 2.6. Full-Length Amplicon Generation of the B646L (p72 Protein)

For all experiments, B646L (p72) amplicon generation reactions were performed using the LongAmp Taq DNA Polymerase (New England Biolabs, Ipswich, MA, USA), according to the manufacturer’s protocol, in a 50 µL reaction using 5 µL DNA as input. The amplification reactions were performed as follows: 94 °C initial denaturation for 30 s, then 30 cycles of 94 °C (30 s), 55 °C (60 s), 65 °C (2 min); with a final extension at 65 °C for 10 min; hold at 10 °C. Following the amplification, the products were purified using Ampure XP beads (Beckman Coulter, Brea, CA, USA) following the manufacturer’s instructions. After purification, the B646L (p72) amplicons were subjected to quality checks using the Tape Station 4200 automated electrophoresis platform using the High Sensitivity DNA 5000 kit (Agilent Technologies, Santa Clara, CA, USA) to confirm the fragment size and quantified using the 1X Qubit dsDNA high sensitivity kit (Thermo Fisher, Waltham, MA, USA).

### 2.7. Library Generation and Nanopore Sequencing

All full-length B646L (p72) amplicons were enzymatically tagged and barcoded using the Rapid Barcoding Kit 24 v14 (SQK-RBK114.24, Oxford Nanopore Technologies (ONT)), which allows multiplexing of up to 24 samples, following the manufacturer’s instructions. Subsequently, libraries were pooled, quantified using the 1X Qubit dsDNA high sensitivity kit (Thermo Fisher, Waltham, MA, USA), and the rapid sequencer adapter was added. Pooled libraries were loaded onto the primed Flongle flow cells (FLG-114, ONT) or primed MinION SpotON flow cell (R10.4.1, ONT) and run on the Nanopore next-generation sequencer GridION (ONT) for 24 (Flongle Flow Cells) or 72 h (R10.4.1 flow cells). All sequencing runs were set up for high-accuracy base calling, with N50 around 1.35 kb to 1.5 kb, with a q-score of 20, with a minimum q-score of 9, as the default for the MinKNOW, software version 23.11.07.

### 2.8. Bioinformatics Analysis Mapping to Best B646L (p72) Reference

A customized Python pipeline was developed to analyze the Nanopore reads. The sequences obtained from the initial read file were aligned using the default parameters of Muscle v3.8.1551 [16], and a preliminary consensus sequence was derived using the Biopython Python library. The specific parameters used for this extraction were as follows: threshold = 0.2 ambiguous = ‘N’ require_multiple = 1 [17].

To identify the most similar match, the preliminary consensus sequence was compared to a carefully curated collection of 55 distinct full-length p72 nucleotide sequences [8] using blastn [18] analysis. The default parameters were employed, except max_hsps was set to 1. The database was composed of the p72 encoded by the following isolates: 26544/OG10 (KM102979), A9_21_3 (OM461370), ASFV_Georgia_2007/1 (FR682468), ASFV_Ken.rie1 (LR899131), ASFV-SY18 (MH766894), BA71V (M34142), Benin_97/1 (AM712239), cro1.2 (AY578690), cro3.5 (AY578691), Davis (MN886934), Dominican_Republic_2 (L76727), DR-1 (L27498), E70 (AY578692), ETH/004 (KT795356), ETH/017 (KT795355), ETH/3a (KT795357), ETH/AA (KT795353), F6 (AY578694), HBNH-2019 (MN207061), JX21 (OM105587), K1 (AY578696), Ken05/Tk1 (KM111294), Ken06.Bus (KM111295), KenIX1033 (NA), Kenya_1950 (AY261360), ker (AY578697), Kimaxia (MN886925), Kitali (MN886937), LIV_5_40 (MN318203), M1 (AY578699), Malawi_Lil-20/1 (AY261361), Mkuzi_1979 (AY261362), Nanuyuki (MN886933), Nigeria_Ni-08_LaOK (MW296951), o1 (AY578701), Pr4 (AY578702), Pr5 (AY578703), Pretoriuskop/96/4 (AY261363), R8 (MH025916), RSA_2_2004 (MN641877), RSA_2_2008 (MN336500), SPEC_57 (MN394630), Spencer (MN886930), TAN/08/Mazimbu (ON409981), Tengani_62 (AY261364), Uganda (NA), Uganda (L27499), UgH03 (EF121429), Uvira_B53 (MT956648), vic (AY578705), Warmbaths (AY261365), Warthog (AY261366), YNFN202103 (ON400500), Za (AY578708), Zaire (MN630494), and Zaire (MW296952). To generate a more refined consensus sequence, the sequences from the initial read file were mapped to the closest reference using the nanopore-optimized parameters of MiniMap2 [19,20]. The mapped reads were then sorted using samtools [21], and variants and gaps were identified using bcftools [21], and bedtools [22], respectively. This process resulted in the creation of a refined consensus sequence, which was subsequently compared to the curated p72 nucleotide dataset as described earlier to determine the closest reference sequence. Subsequently, for each sample, the first 50 Nanopore read files were combined sequentially (e.g., file 1, file 1 + file 2, file 1 + file 2 + file 3). For each file, the reads were mapped against the closest reference sequence identified previously, using the nanopore-optimized parameters of MiniMap2. The consensus sequence for each sample was then extracted using samstools, bcftools, and bedtools following the same methodology as described earlier. The depth of coverage and mapping quality were calculated for each read-alignment for all positions using samtools mpileup [21], with the maximum depth set to unlimited. The closest p72 nucleotide sequence was determined using blastn, as previously described. Finally, graphs were generated using matplotlib [23]. Unless specified in the graph, comparison of different samples was performed using each sample’s first read file generated from Nanopore.

### 2.9. B646L (p72) Alignment and Phylogenetic Tree Construction

Consensus full-length B646L nucleotide sequences generated from the pipeline were aligned in CLC Genomics Workbench 23.0.2 (QIAGEN, Aarhus, Denmark) using the create alignment tool (Gap open cost = 10, Gap extension cost—1, End gap sot = as any other, Alignment mode = Very accurate, Redo alignments = No, and Use fixpoints = No) with the 57 p72 sequences mentioned above plus Bartlett II (MN886935), Caserta (MN886926), Diouroup_II (MN886936), and Salamanca (MN886929). A phylogenic tree was constructed in CLC Genomics using the following parameters of the create phylogenetic tree tool: algorithm = unweighted pair group method with arithmetic mean, distance measure = jukes-cantor, and bootstrap = 1000 replicates.

## 3. Results and Discussion

### 3.1. Primer Design and Selection

**Figure 1:**
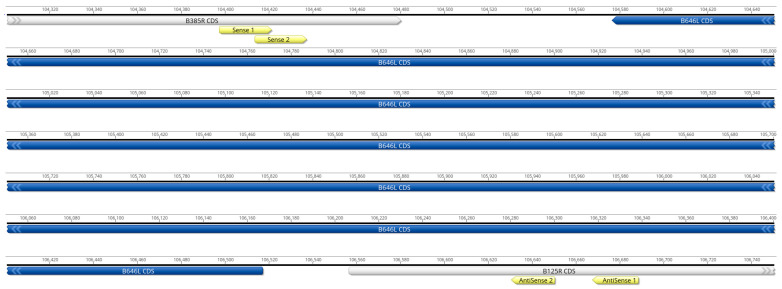
Schematic diagram indicating the position of the B646L (p72) flanking primers. The diagram illustrates the 100% coverage length of the B646L gene; the positions of the S1 and S2, and AS1 and AS2 primers are indicated as yellow arrows, upstream and downstream of the B646L gene.

**Figure 2:**
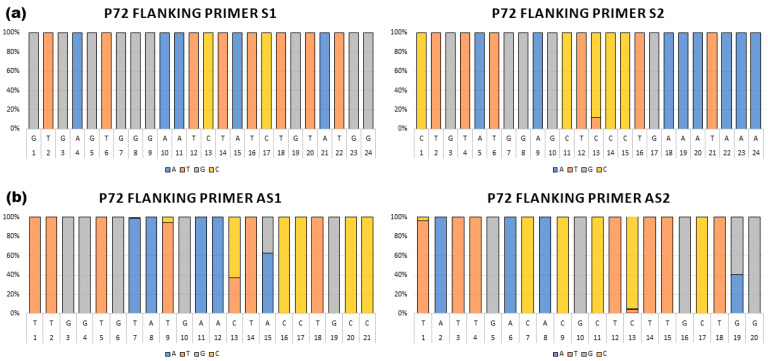
Conservation of sense and antisense primers for the full-length sequence of the B646L (p72) gene. Conservation is depicted as a bar chart, and frequency (percentage) of occurrence of each nucleotide at each position in comparison to 260 full length ASFV sequences is shown. (**a**) Sequence conservation of flanking sense primers S1 and S2; (**b**) Sequence conservation of flanking antisense primers AS1 and AS2. Nucleotide content is shown in blue, orange, gray, and yellow, representing A, T, G, and C, respectively.

A total of four primers, two sense located at positions 139 and 150 nt upstream of the B646L (p72) open reading frame (ORF) and two antisense located at positions 113 and 150 nt downstream of the B646L (p72) ORF, were designed targeting conserved areas upstream or downstream of the B646L gene (Table 1, Figure 1). Each primer sequence was aligned against 260 full length ASFV genome sequences using blastn as described above. Exact matches and the location of SNPs were then identified and quantified. The sense 1 (S1) primer shared 100% nucleotide identity with all 260 aligned ASF sequences, while the sense 2 (S2) primer shared up to 100% nucleotide identity with 229 out of all 260 ASF aligned sequences, and 95.8% identity with 31 ASF isolates due to one nucleotide position in the middle of the primer. The antisense 1 (AS1) primer displays sequence conservation of 100% identity with 145 isolates, 90.5% with 97 isolates due to 3 internal nucleotides, and 95.2% identity with 18 isolates due to a single internal mismatch. When the antisense 2 (AS2) primer was evaluated, the designed primer showed 100% identity with most of the ASF sequences (242 ASF isolates), except for some isolates that showed 85% (7 isolates), 90% (97 isolates) or 95.0% (11 isolates), due to three (5′ and 3′ end), two (3′ end), or one nucleotide mismatches (internal), respectively (Figure 2, Supplemental Material Table S1). Our analysis demonstrated that the Eastern Africa isolates make up most of the variants, with most of the isolates representing genotypes I and II showing 100% identity (Supplementary Material Table S1). Therefore, we are continuing to evaluate these sets of primers for the rapid detection and characterization of ASF using the full-length B646L (p72) sequence.

### 3.2. Evaluation of the Set of Primers to Generate Full-Length B646L (p72) Amplicons

**Figure 3:**
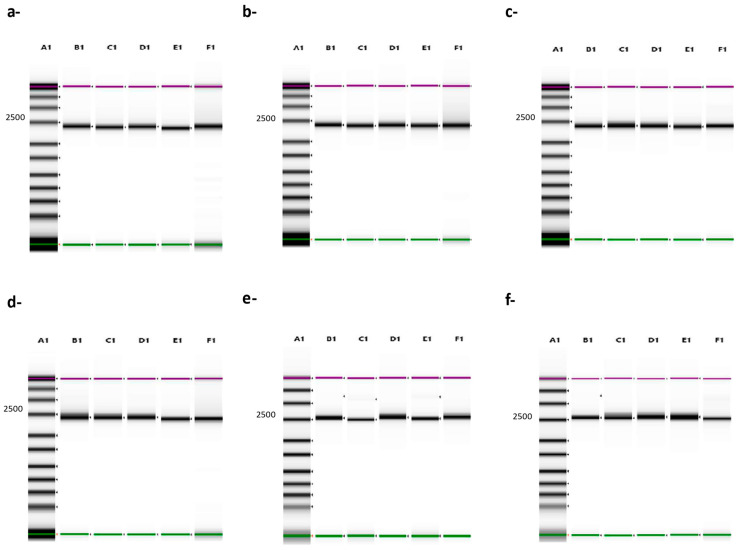
Tape Station electrophoresis of p72 amplicon products out of six different, diverse ASFV strains (**a-**) Lisbon/60, (**b-**) Georgia 2007/1, (**c-**) Malawi Lil-20/1, (**d-**) Pretoria-4/1, (**e-**) Killean III, (**f-**) Kimakia-64), using the primers as individually combined or as a primer pool. A1: ladder, B1: primers S1 + AS1, C1: primers S1 + AS2, D1: primers S2 + AS1, E1: S2 + AS2, F1: pooled primers.

All four primers were evaluated in all possible combinations with a panel of six different diverse ASF strains (Lisbon/60, Georgia 2007/1, Malawi Lil-20/1, Pretoria-4/1, Killean III, and Kimakia-64). All primer sets amplified the full-length sequence of B646L (p72), producing an amplicon product of about 2300 bp, which was confirmed by Tape Station electrophoresis (Figure 3). The *B646L* (p72) amplicons were used to generate libraries using the Rapid Barcoding Kit (ONT) and ran on a Nanopore GridION sequencer. For all the strains, 100% of the B646L (p72) was resolved within a few minutes of starting the runs, with a depth of almost 10^3^ X.

**Figure 4:**
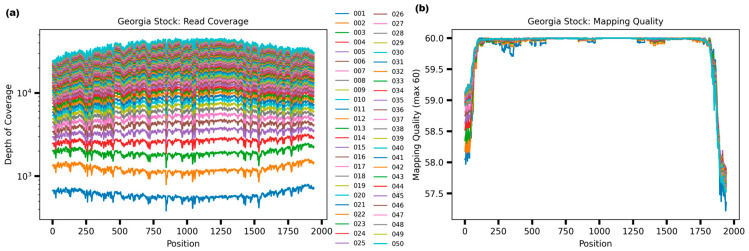
Depth of coverage with accumulative fastq files when ASFV Georgia 2007/1 was tested. (**a**) Read coverage vs. depth of coverage after accumulative number of cycles (**b**) Mapping quality after accumulative number of cycles.

**Figure 5:**
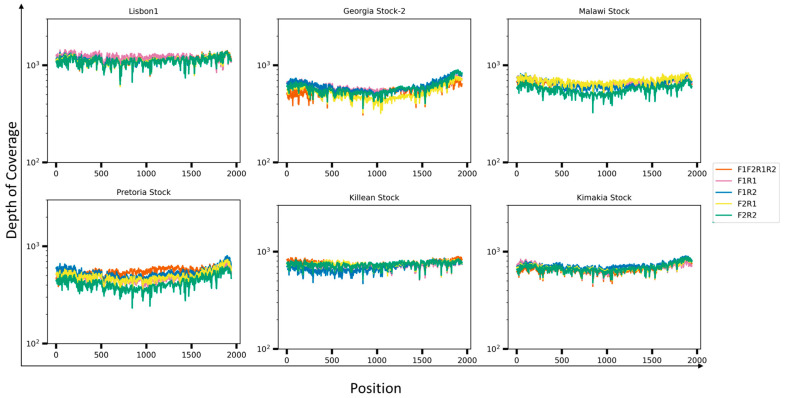
Depth of coverage of fastq Nanopore reads mapped against the full-length B646L (p72) amplicon amplified from the six ASFV strains (Lisbon/60, Georgia 2007/1, Malawi Lil-20/1, Pretoria-4/1, Killean III, and Kimakia-64) using the indicated sets of primers or primer pool. Primer combinations are shown in pink (F1R1), blue (F1R2), yellow (F2R1), and green (F2R2), and red (pooled primers).

Previously we demonstrated a percent identity between 90–100% when comparing the primer product sequences to 260 ASF isolates; therefore, to increase the possibility of detecting a broad range of ASF isolates and different genotypes, all primers were pooled. For these experiments, the same six ASF strains were evaluated by comparing the full-length B646L (p72) amplicon products obtained with the pooled primers with the products obtained when all four different combinations were tested. Primer combinations were as follows: S1 + AS1, S1 + AS2, S2 + AS1, and S2 + AS2. All PCR runs produced an amplicon product of approximately 2300 bp with individual combinations, and similarly, when the pooled primers were used, the same expected size amplicon was produced and confirmed by Tape Station electrophoresis (Figure 3). Full-length B646L (p72) amplicon products were used for library preparation using the Rapid Barcoding Kit v14 (ONT), with an input of 50 ng per sample, as recommended by the manufacturer when more than 4 samples are multiplexed. Preliminary evaluation of the depth of coverage when ASF Georgia 2007/1 was run showed that when using only one fastq file, there was almost 10^3^ X depth with 100% coverage of the whole B646L (p72) gene, increasing up to 10^4^ X with accumulative fastq files, with a mapping quality above 50 (Figure 4, representative run with ASF Georgia 2007/1, showing only up to 43 fastq files). One advantage of Nanopore sequencing is the ability to generate data in real-time, facilitating the rapid analysis, which is critical during an outbreak investigation. Therefore, we focused on the evaluation of only the first 50 fastq files for rapid and real-time characterization of the ASFV genotype, so that there was no need to wait for the runs to be completed (72 h). When pooled primers were used, the B646L (p72) gene was fully resolved a few minutes after the run started, with a depth between 10^2^ X–10^3^ X, in agreement with the data obtained when individual combinations of the primer sets were tested with the six ASFV strains (Figure 5). Last, ASFV Lisbon/60 and Georgia 2007/1 were selected as the most representative strains for genotypes I and II, respectively, to evaluate the consitency of the amplicons and libraries to fully resolve the B646L (p72) gene a few minutes after the runs started. For this experiment, three independent extractions for each ASFV strain, Lisbon/60 and Georgia 2001/7, were processed for amplicon and library preparation, and pooled libraries were sequenced on the GridION platform. All three replicates for both ASFV strains showed a 100% resolution of the B646L (p72) gene, with similar coverage and depth between all three replicates per sample, just after one fastq file was analyzed. Overall, the use of the primer pool for rapid detection and characterization is recommended to achieve high-resolution sequence data, with the objective to mitigate against sequence diversity, increasing the chances to obtain the full length B646L (p72) gene sequence covering most of the genotypes, with a depth of almost 10^3^ X within a few minutes of starting the run, and achieving consistency. The capability to obtain data in real-time allows for rapid analysis, which is critical for a quick identification and diagnosis to identify strains of ASFV that may be circulating during an outbreak.

### 3.3. Genotyping ASFV by Using Full-Length Sequence of the B646L (p72) Gene

**Figure 6:**
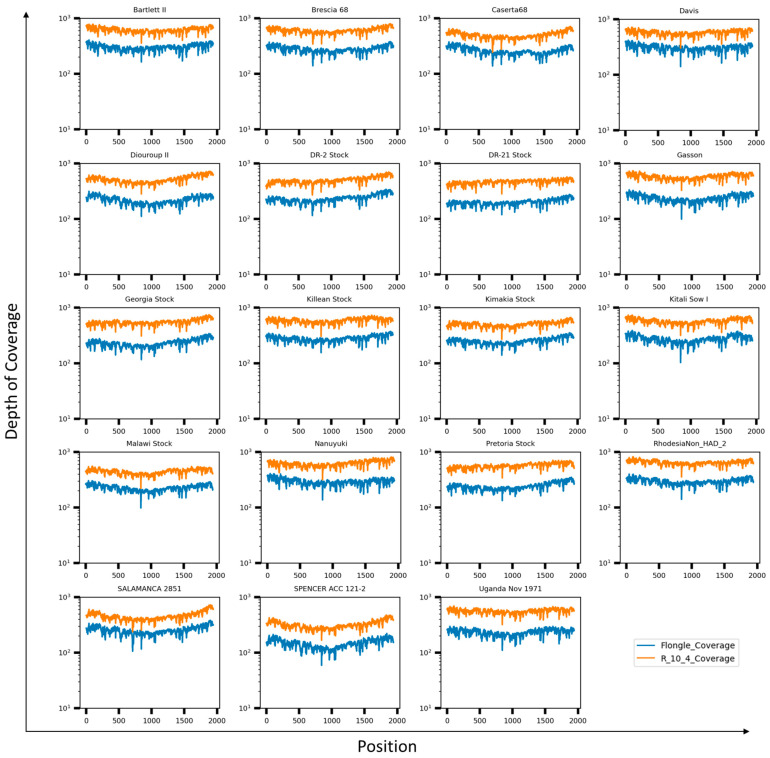
Depth of coverage of Nanopore reads generated on MinION SpotON R10.4.1 (shown in orange) and Flongle FLG-114 (shown in blue) flow cells assessed against a full-length B646L (p72) amplicon amplified from pooled primers and the indicated ASFV strains.

Afterward, we evaluated the capacity of the primer pool for sequencing and genotyping a set of genetically distinct ASFV isolates. ASFV strains used for this experiment are listed in Table 2 and were obtained from the USDA-NVSL-FADDL, Plum Island Biorepository. Amplicons of the B646L (p72) gene were generated using the primer pool and evaluated by Tape Station to confirm the correct size fragment had been generated. Concentrations were determined by quantitating on a Qubit device, and libraries were prepared using inputs of 50 ng total quantity of amplicon per sample. Pooled libraries of six to seven barcoded amplicons were combined, loaded onto the primed MinION SpotON flow cell, R10.4.1 (ONT), and sequenced on the Nanopore GridION platform. For all the ASFV strains used, the whole B646L (p72) gene was fully resolved within minutes after the runs started, with a depth of over 10^2^ X for all isolates, allowing for characterization of each of the isolates by p72 genotype (Figure 6).

**Figure 7:**
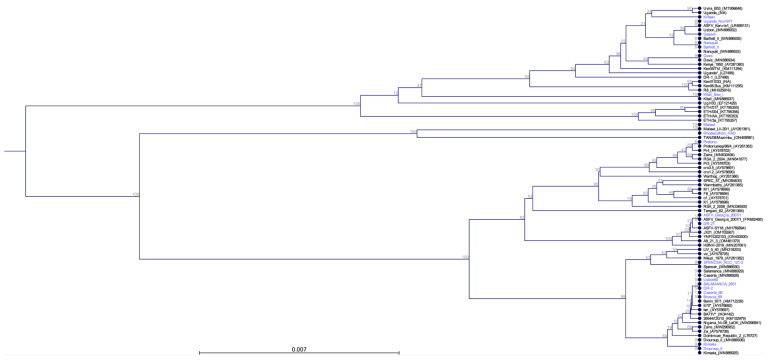
Phylogenetic analysis of all nineteen ASFV strains full-length B646L (p72) nucleotide sequences (shown in blue). The phylogenic tree was constructed in CLC Genomics using the following parameters of the create phylogenetic tree tool: algorithm = unweighted pair group method with arithmetic mean, distance measure = jukes-cantor, selected alignment = all_ref_and_sample_p72 alignment, and bootstrap = 1000 replicates. The phylogenetic tree includes all B646L sequences available at GenBank for reference (shown in black).

Furthermore, we evaluated the performance of the Flongle Flow Cells, FLG-114 (ONT), as a more cost-effective approach, with a decreased turnaround time and the capability to multiplex. To assess the utility of the FLG-114, the same amplicon products obtained for the same nineteen ASFV strains were evaluated using 50 ng of amplicon per sample as input for library preparation. Samples were multiplexed similarly as for the R10.4.1 flow cells, loaded onto the primed FLG-114, and sequenced on the Nanopore GridION platform for comparison. Our results showed that when multiplexing samples and loading them on FLG-114 flow cells, all samples could be resolved for the whole B646L gene with a depth of coverage over 10^2^ X, similar to what was observed when R10.4.1 flow cells were used. Our data indicated that the full-length B646L (p72) sequences obtained by multiplexing samples on Nanopore allows for resolution of the whole B646L gene in just a few minutes after the runs were started, with a rapid characterization of each isolate by their corresponding genotype. Additionally, implementation of FLG-114 Flongle flow cells maintains the performance of resolution with high depth and 100% coverage, allowing a short turnaround time for a quick genotyping characterization, reduces the cost per flow cell by up to 90%, and maintains the multiplexing capability. Examination of the phylogenetic tree for all nineteen ASFV B646L (p72) sequences resulted in classification into their corresponding cluster based on the genotypes (Figure 7), and data was consistently independent of using sequence reads obtained from the R10.4.1 or FLG-114 flow cells.

### 3.4. Resolution of the B646L (p72) Gene in Whole-Blood Samples from ASFV Experimentally Infected Pigs

**Figure 8:**
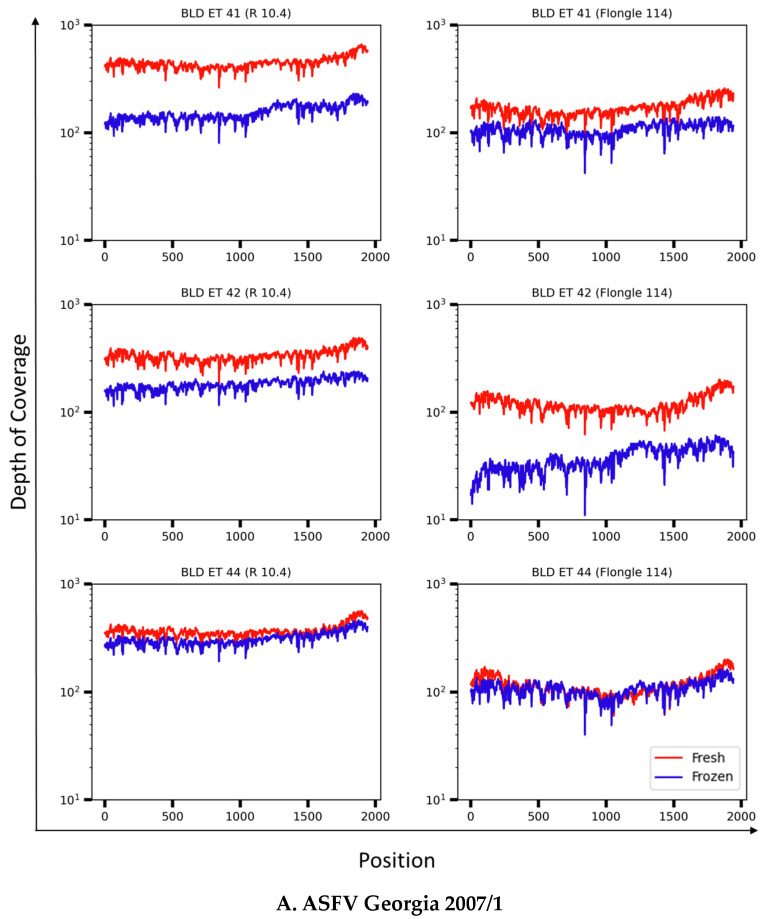

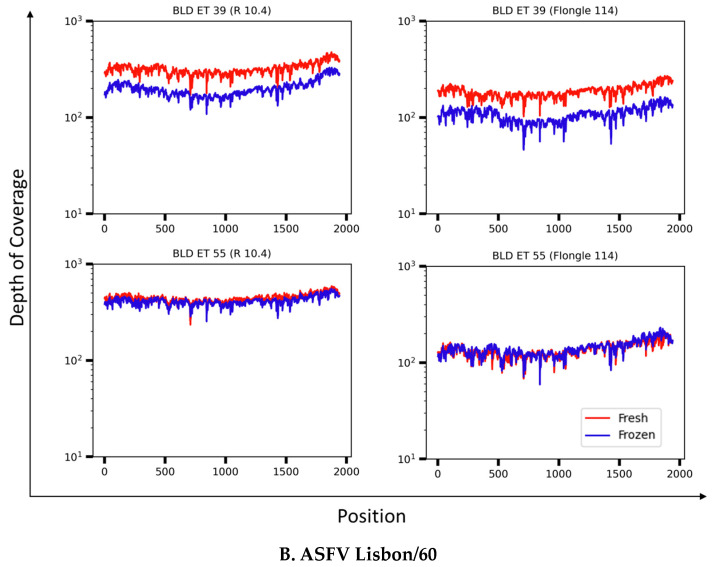
Read and depth coverage of B646L (p72) synthesized using the primer pool with fresh (red) and frozen (blue) whole-blood samples from pigs experimentally infected with ASFV Georgia 2007/1 (**A**) or Lisbon/60 (**B**) were compared along with performance of MinION SpotON R10.4.1 (**left**) or Flongle FLG-114 (**right**) flow cells. BLD ET: blood pig ear tag.

We next evaluated how the primer pool performs when blood samples were used as a reference sample, since infection with ASFV is related to a high viral load in blood that can be up to 10^7^ to 10^9^ 50% hemadsorption dose (HAD_50_)/mL [24]. For this experiment, we conducted a comparison between fresh and frozen blood samples collected from pigs experimentally infected with ASFV Lisbon/60 or Georgia 2007/1, as the most common strains representing genotypes I and II, respectively (Table 3). The comparison of fresh versus frozen samples was done because there are times when samples might not be processed immediately, or when it may be necessary to revisit historical samples for further characterization and comparison to current outbreak-associated strains. Blood samples were taken during the acute phase of infection, when clinical signs of the disease manifest. Nucleic acid extracts from fresh and frozen blood samples were amplified by qPCR and demonstrated similar Ct values between both treatments (Table 3). Preliminary experiments were done with one fresh blood sample per ASF strain, Lisbon/60 or Georgia 2007/1, using all individual primer set combinations. As previously observed when the viral stocks corresponding to the same strains were tested, 100% resolution of the B646L (p72) gene was obtained with a similar depth of coverage independent of the primer set used, just after one fastq file was analyzed; therefore, all further evaluations of blood samples were performed using the primer pool to generate B646L (p72) amplicons for library preparation. Libraries were loaded multiplexed and sequenced on the Nanopore GridION platform using R10.4 flow cells or FLG-114 flow cells for further comparison. The fragment size of the amplicon product was confirmed by Tape Station, with a size of approximately 2300 bp as the expected product. All samples, independent of the treatments, fresh or frozen, resulted in a 100% read coverage with enough depth of 10^2^ to 10^3^ X to be easily genotyped (Figure 8). Additionally, when both flow cells were compared, as previously observed, an increase in depth of coverage was observed when R10.4.1 flow cells were used. This result was expected due to the high throughput, large number of pores, and longer sequencing time. Still, the FLG-114 flow cells allowed 100% coverage and resolution of the full-length B646L (p72) gene in a short time, with most samples resulting in a depth of 10^2^ X or above. In conclusion, our results demonstrated consistency when samples were tested fresh or frozen, albeit variations in depth for full resolution, but with enough depth to determine the correct genotypes.

## 4. Conclusions

Our preliminary analysis of all publicly available ASFV sequences demonstrated that these sets of primers were highly conserved between different isolates. Although they were designed using genotype II, the current pandemic strain in Asia, Africa, Europe, and the island of Hispaniola, the primers were able to amplify from the majority of the reported genotypes currently causing outbreaks worldwide. Still, as NGS becomes more common and more sequences of ASFV isolates become available, it is possible that a re-design of alternative primers could be taken into consideration based on new emerging circulating ASFV strains. In this study, the pooling of primers showed an increased ability to detect many ASFV isolates and different genotypes, and adding primers from new emerging strains or changing one of the primers could allow for the adaptation of this method to new emerging strains of ASFV not covered in this study.

Here we show that with all twenty ASFV strains tested in this work, the B646L (p72) sequences matched with the predicted genotypes as determined using the traditional genotyping method using the nucleotide sequence of the C-terminal region of p72 [7], or by using the whole B646L (p72) sequence for the new characterization of genotypes [8], allowing for rapid detection and characterization by genotypes using both methods for ASFV in real-time using Nanopore technology. One of the advantages of Nanopore sequencing is the ability to generate data in real-time, thus facilitating the rapid analysis that is critical during an outbreak investigation. In this report, there was no difference in the ability to sequence fresh or frozen samples, allowing for the option to test fresh samples in the field or ship frozen samples to a diagnostic laboratory.

However, even with the decreasing costs of Nanopore sequencing, the costs for consumables are still high for testing in many countries where ASF outbreaks are currently occurring. To help reduce the cost, we evaluated FLG-114 Flongle flow cells versus MinION SpotON R10.4.1 flow cells. The FLG-114 Fongle flow cells performed as well as MinION SpotON R10.4.1 flow cells, with rapid turnaround of ASFV identification, with enough depth and complete coverage of the B646L (p72) ORF within only a few minutes of starting the sequencing runs. In addition to the implementation of FLG-114 Flongle flow cells to reduce the cost of sequencing samples, we determined that the capability to multiplex at least 7–10 samples is possible; and, with further development, it could be possible to test up to 24 or more different barcodes per flow cell in real time, which could further reduce the cost.

As ASF vaccines are starting to be approved and used (e.g., Vietnam), it is important that vaccines be used to correctly match outbreak strains. The rapid sequencing and characterization presented here could meet those needs by being able to rapidly characterize outbreak strains. Although here we present the rapid sequencing of B646L (p72) from ASFV, we have previously reported the use of Nanopore technology to rapidly provide full-length sequencing of ASFV [25,26]), which could also be a powerful diagnostic tool; or this system could also be adapted to identify and characterize ASFV by targeting multiple genes such as the PACT ((p72 [P], p54 [A], CVR of the 9RL-ORF [C], and TK [T]) method [27].

## Figures and Tables

**Table 1:** Primer sequences designed for full-length B646L gene amplicon generation.

Target B646L Gene	Sequence (5′–3′)	Position (bp)
Sense 1	GTGAGTGGGAATCTATCTGTATGG	150 nt upstream
Sense 2	CTGTATGGAGCTCCCTGAAATAAA	139 nt upstream
Antisense 1	GGCAGGTAGTTCATACACCAA	150 nt downstream
Antisense 2	CCAGCAAGAGCGTGTCAATA	113 nt downstream

**Table 2:** Summary of ASFV isolates used to evaluate B646L (p72) gene.

ASF Strain	Country of Origin	Genotype (Historic)	Genotype	ASF rt-PCR (Ct)	GenBank Accession No.
Georgia/2007	Georgia	II	2	21.68	FR682468
DR-21	Dominican Republic	II	2	24.86	n.a.
Killean III	Kenya	X	9	20.02	n.a. ^1^
Kimakia-64	Kenya	I	1	20.29	MN886925
Malawi Lil-20/1	Malawi	VIII	8	18.17	AY261361
Pretoria-4/1	South Africa	XX	2	22.79	AY578702
Lisbon/60	Portugal	I	1	20.55	n.a. ^2^
Nanuyuki	Kenya	X	9	23.9	MN886933
Kitali, Sow I	Kenya	IX	9	22.73	MN886937
Gasson	Kenya	X	9	21.9	n.a. ^3^
Spencer	South Africa	n.a	1	17.28	MN886930
Salamanca	Spain	I	1	19.67	MN886929
Davis	Kenya	X	9	21.16	MN886934
Bartlett II	Kenya	X	9	20.91	MN886935
Rhodesia non-HA	South Africa	VIII	8	20.67	n.a.
Caserta 68	Italy	I	1	22.3	MN886926
Brescia 68	Italy	I	1	23.79	n.a.
Uganda 1971	Uganda	X	9	17.73	n.a.
Diouroup II	Senegal	I	1	22.36	MN886936
DR-2	Dominican Republic	I	1	23.03	n.a. ^4^

^1^ Only a partial sequence exists (AY351531). ^2^ There is a GenBank Accession No. for Lisbon; however, it is not in agreement with the corresponding genotype I. ^3^ Only a partial sequence exists (AY351529). ^4^ There is a GenBank Accession No. for DR 2 (L76727); however, the sequence is slightly incorrect and leads to a change in an amino acid. n.a.: non-available.

**Table 3:** Summary of ASFV whole blood samples to evaluate the competency of B646L (p72) amplicons when using all primer combinations vs. the primer pool and sequenced on a Nanopore next-generation GridION sequencer using R10.4.1 flow cells vs. FLG-114 flow cells. Samples correspond to five pigs experimentally infected, as described in Materials and Methods, with ASFV Georgia 2007/1 or Lisbon/60. I.D.: pig ID.

ASFV Strain	ID	d.p.i.	qPCR (Ct) (Fresh)	qPCR (Ct) (Frozen)
Georgia 2007/1	41	6	20.3	21.15
Georgia 2007/1	42	6	20.78	20.83
Georgia 2007/1	44	6	18.8	18.81
Lisbon/60	55	7	19.46	20.55
Lisbon/60	39	9	20.18	20.65

## Data Availability

All data are included in the manuscript. All ASFV reference genomes were obtained from GenBank, and the GenBank accession number of each sequence has been provided in Supplementary Table S1.

## Supplementary Materials

The following supporting information can be downloaded at: https://www.mdpi.com/article/10.3390/v16101522/s1, Supplementary Table S1: Conservation of sense and antisense primers for the full-length sequence of the B646L gene.
